# Supplementary material for: Effects of Acute Exercise on Cognitive Flexibility in Young Adults with Different Levels of Aerobic Fitness
Source: Int J Environ Res Public Health. 2022 Jul 26;19(15):9106. doi: 10.3390/ijerph19159106 (PMC9331115; doi:10.3390/ijerph19159106)
Supplement: Supplementary file 1 [file ijerph-19-09106-s001.zip › ijerph-1720651-supplementary.pdf]

Table S1: Reaction time and accuracy for high-fitness group on the more-odd shifting task before (pre) and after (post) for the four interventions (10 min HIIE, 20 min HIIE, 20 min MICE and control condition). Data are shown as mean ± standard deviation (M ± SD).

High-fitness		Non-switching				switching			
		10-min HIIE	20-min HIIE	20-min MICE	Control	10-min HIIE	20-min HIIE	20-min MICE	Control
RT (ms)	Pre	543.02 ± 83.55	542.85 ± 82.97	541.04 ± 77.61	533.86 ± 80.19	675.21 ± 122.22	681.18 ± 110.52	656.08 ± 117.17	651.08 ± 107.40
	Post	523.98 ± 70.28	555.90 ± 77.64	521.34 ± 82.72	538.62 ± 96.92	649.83 ± 104.11	618.47 ± 96.69	665.63 ± 189.34	638.94 ± 113.56
Accuracy (%)	Pre	93.66 ± 4.78	94.13 ± 4.04	93.09 ± 5.34	92.71 ± 4.92	89.96 ± 5.17	90.15 ± 6.78	88.54 ± 6.43	89.87 ± 5.90
	Post	91.95 ± 6.35	93.28 ± 4.82	93.66 ± 4.17	92.61 ± 5.46	90.72 ± 5.61	90.91 ± 5.43	89.68 ± 6.27	89.87 ± 5.30

Notes: HIIE: high-intensity interval exercise; MICE: moderate-intensity continuous exercise.

Table S2: Reaction time and accuracy for low-fitness group on the more-odd shifting task before (pre) and after (post) for the four interventions (10 min HIIE, 20 min HIIE, 20 min MICE and control condition). Data are shown as mean ± standard deviation (M ± SD).

Low-fitness		Non-switching				switching			
		10-min HIIE	20-min HIIE	20-min MICE	Control	10-min HIIE	20-min HIIE	20-min MICE	Control
RT (ms)	Pre	552.94 ± 83.15	569.91 ± 92.85	557.68 ± 72.83	567.31 ± 84.55	715.40 ± 128.63	719.41 ± 136.34	721.24 ± 126.92	707.68 ± 114.30
	Post	546.66 ± 84.04	555.89 ± 77.64	530.07 ± 78.46	556.99 ± 86.54	663.09 ± 126.89	687.64 ± 130.23	678.57 ± 122.84	705.88 ± 133.08
Accuracy (%)	Pre	93.56 ± 5.58	93.56 ± 5.58	93.75 ± 5.24	93.75 ± 4.82	91.67 ± 5.89	93.18 ± 4.97	91.29 ± 4.26	91.95 ± 4.82
	Post	92.23 ± 6.45	92.61 ± 4.61	92.61 ± 4.61	94.03 ± 4.65	90.44 ± 6.25	89.58 ± 6.66	91.86 ± 6.25	89.87 ± 6.58

Notes: HIIE: high-intensity interval exercise; MICE: moderate-intensity continuous exercise.

Table S3: Switch cost for high and low fitness groups on the more-odd shifting task before (pre) and after (post) the four interventions (10 min HIIE, 20 min HIIE, 20 min MICE and control condition). Data are shown as mean ± standard deviation (M ± SD).

	High-fitness				Low-fitness			
	10-min HIIE	20-min HIIE	20-min MICE	Control	10-min HIIE	20-min HIIE	20-min MICE	Control
Pre	132.19 ± 65.47	125.85 ± 52.14	115.04 ± 82.28	117.22 ± 63.04	162.46 ± 81.75	149.49 ± 80.25	163.56 ± 87.50	140.36 ± 66.25

Post	125.85 ± 52.14	95.42 ± 54.68	106.46 ± 64.84	100.32 ± 72.54	116.44 ± 70.39	131.74 ± 90.56	139.75 ± 85.93	148.89 ± 85.46

Notes: HIIE: high-intensity interval exercise; MICE: moderate-intensity continuous exercise.
